# Uterine Inflammatory Myofibroblastic Tumor Mimicking Myoma Treated With Gonadotropin-Releasing Hormone Therapy: A Case Report

**DOI:** 10.7759/cureus.84722

**Published:** 2025-05-24

**Authors:** Kenji Yorita, Koki Hirano, Kimiko Nakatani

**Affiliations:** 1 Department of Diagnostic Pathology, Japanese Red Cross Kochi Hospital, Kochi, JPN; 2 Department of Obstetrics and Gynecology, Japanese Red Cross Kochi Hospital, Kochi, JPN; 3 Department of Radiology, Japanese Red Cross Kochi Hospital, Kochi, JPN

**Keywords:** inflammatory myofibroblastic tumor, leiomyoma, pathology, radiology, uterus

## Abstract

Inflammatory myofibroblastic tumors (IMTs) of the uterus are a rare entity that can be challenging to distinguish from leiomyomas, both radiologically and pathologically. No previous reports have documented the response of uterine IMT to gonadotropin-releasing hormone (GnRH) therapy. A 47-year-old Japanese woman presented to our hospital with excessive menstruation and uterine prolapse. Magnetic resonance imaging (MRI) revealed four well-demarcated uterine masses, with the largest measuring 81 mm. Myomas were suspected. Oral GnRH therapy was initiated to relieve the symptoms and reduce the preoperative volume. After four months, follow-up MRI showed a reduction in the largest mass to 62 mm and increased hyperintense areas on T2-weighted images. At six months, the patient underwent laparoscopic hysterectomy and colporrhaphy. Histopathological examination revealed that the largest tumor was an IMT with abundant myxoid matrix, positive for anaplastic lymphoma kinase (ALK), and ALK gene rearrangement. The remaining three masses were diagnosed as usual-type leiomyomas. This is the first reported case of uterine IMT treated with GnRH therapy, which resulted in notable tumor shrinkage. This case highlights both the potential therapeutic approach for uterine IMT and the diagnostic difficulty in distinguishing it from leiomyomas preoperatively.

## Introduction

Inflammatory myofibroblastic tumors (IMTs) are mesenchymal neoplasms occurring at various sites, but rarely in the uterus (<100 cases reported) [1]. Uterine IMT is characterized by the proliferation of myofibroblastic and fibroblastic spindle cells accompanied by a myxoid matrix and inflammatory infiltrates [1]. Most IMTs are immunohistochemically positive for anaplastic lymphoma kinase (ALK), which typically correlates with ALK gene alterations [1]. Uterine IMT can mimic other uterine tumors, particularly leiomyomas, both clinically and pathologically. Although uterine IMTs are often considered benign, they rarely extend or recur outside the uterus [2,3]. In a 2017 review article, of 53 cases of uterine IMT with follow-up data, 46 cases (87%) were free of disease, and four cases (9%) were reported to have died of the disease [4]. Understanding the radiological and pathological features of such tumors is crucial for proper diagnosis. However, imaging findings that are considered characteristic of uterine IMT have not been reported [5], while rare cases pathologically misdiagnosed as uterine leiomyomas have been reported [6]. The treatment of uterine IMT involves surgical resection in >90% of cases, although there are some reports of reduction with the use of ALK inhibitors in unresectable cases [4,7]. However, to our knowledge, no cases of hormone therapy have been reported.

This case report describes a 47-year-old Japanese woman with uterine IMT who was clinically misdiagnosed with uterine myoma and treated with gonadotropin-releasing hormone (GnRH) therapy. To our knowledge, this is the first reported case of uterine IMT treated with GnRH therapy. This case highlights the radiopathological diagnosis of uterine IMT and provides novel insights into the potential effectiveness of GnRH therapy for reducing the size of uterine IMT.

## Case presentation

Clinical history

A 47-year-old woman with a history of four pregnancies, three spontaneous abortions, and one full-term vaginal delivery was referred to our hospital for treatment of uterine corpus tumors. The patient’s family history was unremarkable. At the age of 46 years, she was pointed out two uterine masses, suggesting myomas, by her previous gynecologist and followed up because of the absence of anemia. Nine months after her first visit to her previous doctor, she was referred to our hospital for treatment of the uterine masses following menorrhagia and uterine ptosis. Vital signs were unremarkable. On bimanual pelvic examination, the uterus was enlarged to approximately 10-week gestational size, with no tenderness. Blood tests revealed no evidence of anemia.

Imaging, clinical diagnosis, and treatment

**Figure 1:**
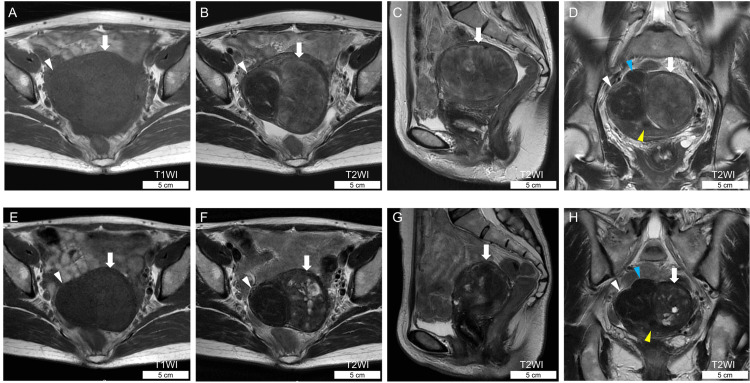
Magnetic resonance images of the uterine IMT. (A,B,E,F) Horizontal sections. (C,G) Sagittal sections. (D,H) Coronal sections. Scale bars are included in the images (A-D) MR images obtained before GnRH antagonist therapy. The uterine IMT (indicated by arrows) exhibits isointensity on T1WIs (A) and heterogeneous signal intensity on T2WI (B-D). The second-largest mass (leiomyoma) (indicated by white arrowheads), the third mass (indicated by blue arrowhead), and the fourth mass (indicated by yellow arrowhead) demonstrate low intensity on T1- (A) and T2- (B-D) WIs. (E-H) MR images obtained four months after the initiation of GnRH antagonist therapy. The uterine IMT (indicated by arrows) appears reduced in size and shows increased hyperintensity on T2WI (F-H) compared to pretreatment images. The second-largest leiomyoma (indicated by white arrowheads) appears slightly decreased in size. The other two leiomyomas (indicated by blue and yellow arrowheads) appear unchanged MR: magnetic resonance; GnRH: gonadotropin-releasing hormone; IMT: inflammatory myofibroblastic tumor; WI: weighted image

Chest radiography revealed no abnormalities. Transvaginal ultrasonography confirmed a uterine mass of 8 cm (graphical data unavailable), and magnetic resonance imaging (MRI) showed four demarcated masses with maximum diameters of 8.1, 5.2, 2.8, and 1.4 cm, respectively, in the uterine corpus (Figures 1A-1D). No bilateral abnormalities were observed in the uterine adnexa. Uterine myomas were clinically diagnosed. Since the patient wished to have her uterine masses removed and uterine ptosis improved, the clinical plan was to perform a laparoscopic hysterectomy and colporrhaphy after six months of treatment with a GnRH receptor agonist (relugolix, 40 mg once daily). Four months after oral administration, MRI showed that the maximum size of the four uterine lesions was 6.2, 4.5, 2.8, and 1.4 cm, with the largest two having a reduction effect with GnRH therapy (Figures 1E-1H). The largest tumor showed increased hyperintensity on T2-weighted images (T2WIs) (Figures 1F-1H). No peritoneal dissemination was observed during surgery.

Pathological findings

**Figure 2:**
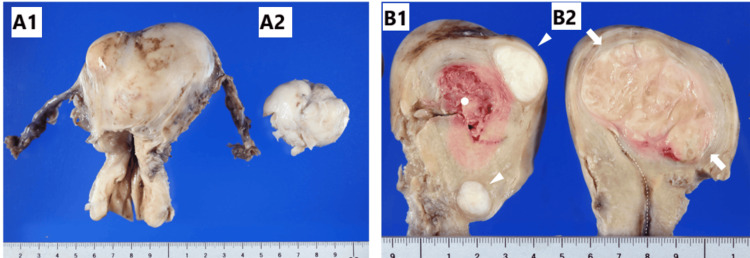
Gross findings of a uterine IMT and three usual-type uterine leiomyomas Resected uterus (A1) and the second-largest mass (leiomyoma, A2). The second largest mass seen detached from the uterus during extraction of the excised uterus through the vaginal route. A uterine IMT (indicated by arrows in B2) appears as a well-demarcated solid intramural mass with a color similar to that of the background uterine myometrium. The third- and fourth-largest tumors (leiomyomas, indicated by arrowheads in B1) are well-demarcated, solid, and white. The dotted line marks the endometrium, while the white dot (B1) indicates the site where the second-largest mass was located. The scale bars represent images IMT: inflammatory myofibroblastic tumor

Macroscopically, four demarcated masses were found in the formalin-fixed, excised uterus, with the largest mass measuring 6.2 × 4.5 × 4.3 cm and the second largest mass measuring 4.5 × 3.5 × 3.0 cm (Figure 2). The largest mass was grayish in color, similar to the surrounding myometrium, whereas the other three masses were white (Figure 2).

**Figure 3:**
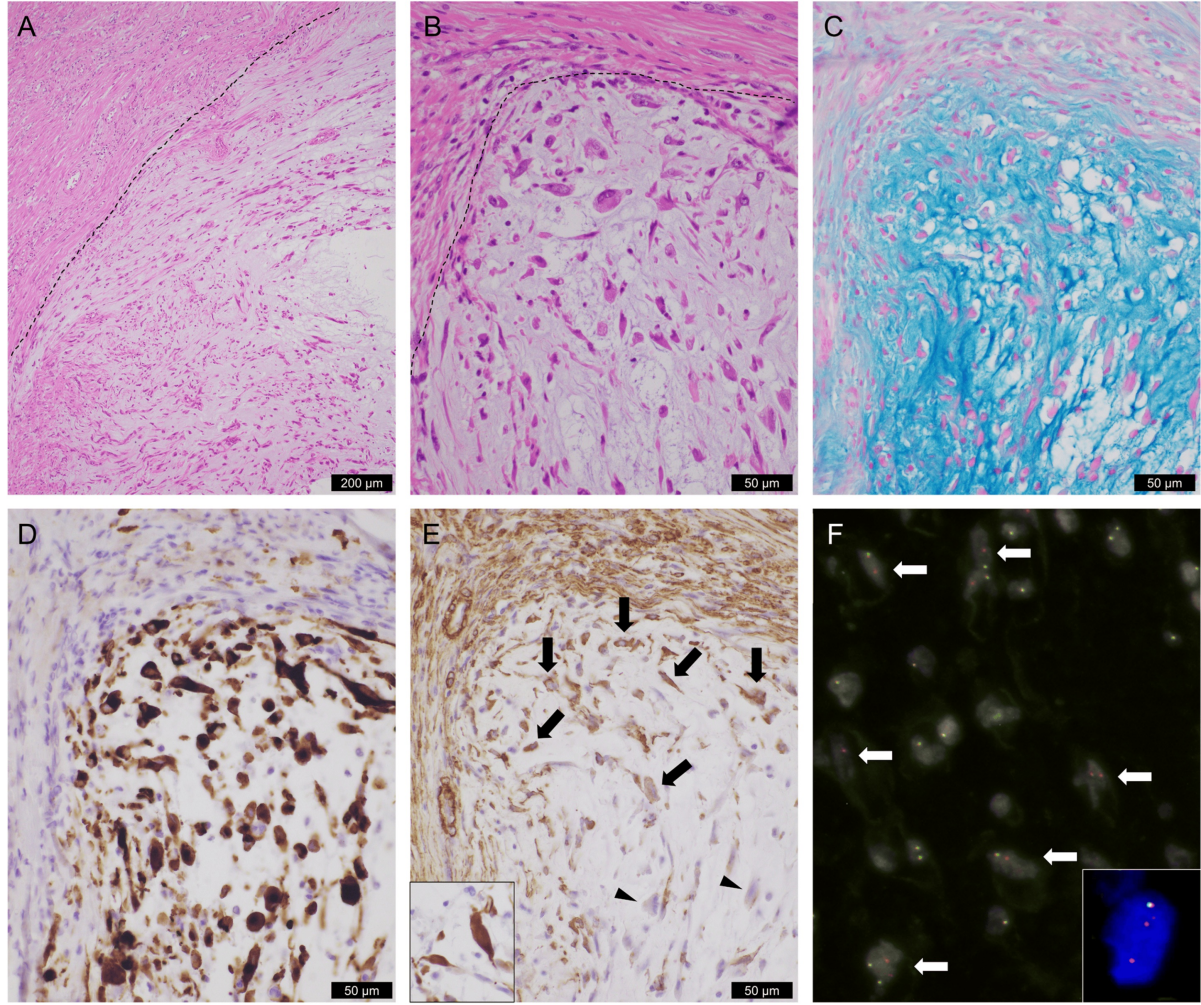
Histological, immunohistochemical, and genetic result of uterine IMT (A,B) Hematoxylin-and-eosin-stained section of a uterine IMT shows that spindle, ovoid, and stellate cells proliferate discohesively within the myxoid stroma. Dotted lines indicate the tumor borders. Myometrial smooth muscle cells are visible on the left and upper sides of the images. (C) Alcian blue-stained section of the uterine IMT reveals an abundant myxoid matrix within the tumor stroma. Immunohistochemically, uterine IMT cells exhibit diffuse positivity for ALK (D, cytoplasmic staining) and focal positivity for alpha-smooth muscle actin (E, arrows). Arrowheads indicate tumor cells negative for alpha-smooth muscle actin. The tumor cells are also positive for desmin (E, inset). (F) Fluorescence in situ hybridization demonstrates ALK gene rearrangement in uterine IMT cells, showing a high rate of split signals (indicated by arrows) in the tumor cells. The inset shows a magnified image of a representative split signal in an IMT cell. (B-F) High-magnification views of the tumor border, revealing nearly the same region. Scale bars are included in the images IMT: inflammatory myofibroblastic tumor; ALK: anaplastic lymphoma kinase

Histologically, the largest mass showed scattered proliferation of atypical cells with spindle-shaped, oval, or stellate morphology, accompanied by abundant myxoid stroma (Figures 3A-3C). Ganglion-like cells were also observed. Infiltrative growth into the myometrium was noted inside the tumor, which also demonstrated that the existing myometrial smooth muscle cells were intermingled within the tumor. Mitoses were rarely observed (~1/2 mm^2^), and neither necrosis nor fibrosis was observed. Lymphoplasmacytic infiltration was focally observed in the tumor. The largest uterine mass was considered to be a mesenchymal tumor with myxoid changes, leading to pathological diagnoses of leiomyoma, myxoid leiomyosarcoma, schwannoma, endometrial stromal tumor, and IMT. Immunohistochemically, the tumor cells were diffusely ALK-positive (Figure 3D), focally positive for α-smooth muscle actin (Figure 3E) and desmin (Figure 3E, inset), and negative for ROS1, SOX10, and CD10. Ki67 labeling index was less than 8%. Fluorescence in situ hybridization (FISH) confirmed ALK rearrangement, as split signals were detected in 144 of 188 tumor cells (77%) using the automated image analysis software Metafer 4 (MetaSystems, Altlussheim, Germany) and the Vysis ALK break-apart FISH probe kit (Abbott Molecular, Des Plaines, IL) (Figure 3F). Therefore, a uterine IMT was considered. No lymphovascular invasion was observed. The other three nodules histologically represented typical leiomyomas that were immunohistochemically negative for ALK.

Postoperative course

The postoperative course was uneventful, and the patient was discharged on postoperative day 3. On postoperative day 21, no extrauterine involvement of the IMT was observed by contrast-enhanced computed tomography. A postoperative follow-up was performed at our hospital, and further observation will be continued. The patient provided informed consent for the publication of this report.

## Discussion

Clinical diagnostic challenges

Here, we report a case of uterine IMT treated with GnRH therapy. Differential diagnoses of uterine IMT include leiomyoma, leiomyosarcoma, and other mesenchymal tumors. No characteristic imaging findings were reported in a review of uterine IMT published in 2021 [5]. In our case, GnRH therapy resulted in a reduction in uterine IMT size and an increase in the hyperintense areas on MRI T2WI, which was possibly due to increased deposition of the myxoid matrix. Fujii et al. reported that uterine IMT shows mixed signal intensity on T2WI, with hyperintensity reflecting the myxoid area [8], one of the histological features of uterine IMT. However, uterine myomas are also known to have edematous or myxoid degeneration. Since relugolix is known to shrink uterine myomas and enhance T2WI signal intensity in shrunken myomas [9], the IMT, in this case, was likely to be clinically misdiagnosed as a uterine myoma. One possible imaging differentiator between uterine IMT and leiomyoma is the presence of irregular tumor borders. Since early reports of uterine IMT showed irregular tumor borders with gross findings [2,3], a uterine IMT might be suspected rather than leiomyoma when irregular tumor borders are observed radiologically. As seen in our case, in a recent report by Pan et al., all 13 cases of uterine IMT revealed well-demarcated lesions [10]. Whether tumor border irregularities are valid for radiological diagnosis of uterine IMT remains to be determined, as there are no reports on tumor border irregularities characteristic of uterine IMT as suggestive radiologic findings.

Pathological diagnostic challenges

In this case, the excised surface of the uterine IMT was vastly different from that of a leiomyoma in terms of color tone after formalin fixation. However, it is difficult to distinguish between the two grossly because it has been reported that all cases of uterine IMT have a typical leiomyoma gross image [11]. Histologically, typical leiomyomas and IMT differ significantly. However, in a review of 1,747 cases pathologically diagnosed as uterine leiomyoma, only five cases (0.3%) were revised to IMT [6], which indicates that morphologically misdiagnosing IMT as leiomyoma rarely occurs. Most of these five cases demonstrated partial histological features of IMT in a retrospective study [6]. In cases of suspected leiomyomas, pathologists should be able to differentiate them from uterine IMTs based on the histological features of uterine IMTs, such as intratumoral myxoid stroma, lymphoplasmacytic infiltration, and infiltrative growth [2]. Accurate diagnosis may be challenging without molecular testing [12]. The latest WHO Health Organization classification of female genital tumors states that the essential diagnostic criteria for uterine IMTs include ALK immunoreactivity, and the desired diagnostic criterion is ALK rearrangement [1]. In this case, ALK rearrangement was confirmed by FISH, as uterine smooth muscle tumors rarely show ALK immunoreactivity [12].

Therapeutic considerations and hormonal responsiveness

There have been no previous reports of GnRH therapy resulting in uterine IMT reduction, although ALK inhibitor-based targeted therapy has been reported to reduce uterine IMTs [4,7]. The MRI and pathological findings of the uterine IMT in our case suggest that GnRH therapy decreased the number of tumor cells and increased the myxoid matrix in the tumor. Relugolix is a GnRH receptor antagonist that suppresses the secretion of gonadotropins and decreases ovarian production of estrogen and progesterone. Uterine IMTs have been reported to be immunohistochemically positive for estrogen receptor (ER) and/or progesterone receptor (PR) [6]. Thus, relugolix may reduce estrogen and progesterone signaling in uterine IMT cells, thereby shrinking the tumor. Additional immunostaining for ER and PR was performed on the IMT in this patient; however, the positivity rate of ER and PR in tumor cells was less than 10%. Considering that GnRH therapy can reduce ER and PR expression in uterine leiomyomas [13], the ER and PR positivity of the uterine IMT in this case might have been reduced by GnRH therapy. GnRH therapy may be effective in shrinking uterine IMTs as well as ALK inhibitors [4,7]. Relugolix has also been reported to act directly on uterine myoma cells to decrease extracellular matrix production, resulting in tumor shrinkage [14]; however, this mechanism does not seem to apply to IMT cells in this case. Since only one case was studied in this case and the preoperative expression of ER and PR in IMT could not be evaluated, we could not conclude that GnRH therapy has a therapeutic effect on IMT. Whether GnRH therapy is effective in uterine IMTs requires the accumulation and analysis of similar cases.

## Conclusions

This case represents the first reported instance of uterine IMT treated with GnRH therapy, which was associated with a reduction in the tumor size. The uterine IMT was confined to the uterus with postoperative imaging evaluation; however, the biological nature of the IMT in this case was not fully investigated owing to the short postoperative follow-up period. The tumor’s well-demarcated borders and MRI response mimicked the typical presentation of leiomyoma, leading to clinical misdiagnosis. While our findings raise the possibility that GnRH therapy may influence IMT behavior, this remains speculative because of the single-patient nature of the observation, and further research is warranted to explore the potential hormonal responsiveness of uterine IMTs. Clinicians and radiologists should be aware that uterine IMT radiologically resembles myomas. Irregular tumor borders may offer a subtle clue, though this is not consistently present. From a pathological perspective, IMT should be considered in the differential diagnosis of uterine mesenchymal tumors when features, such as myxoid stroma, infiltrative growth, or lymphoplasmacytic infiltration, are noted, even focally.
